# Glutamine Uptake via SNAT6 and Caveolin Regulates Glutamine–Glutamate Cycle

**DOI:** 10.3390/ijms22031167

**Published:** 2021-01-25

**Authors:** Nikhil R. Gandasi, Vasiliki Arapi, Michel E. Mickael, Prajakta A. Belekar, Louise Granlund, Lakshmi Kothegala, Robert Fredriksson, Sonchita Bagchi

**Affiliations:** 1 Department of Metabolic Physiology, Institute of Neuroscience and Physiology, University of Göteborg, Box 430, SE-405 30 Göteborg, Sweden; prajakta.belekar.1@gmail.com (P.A.B.); lakshmi.kothegala@gu.se (L.K.); 2 Department of Pharmaceutical Biosciences, Molecular Neuropharmacology, Uppsala University, SE-751 23 Uppsala, Sweden; valia_arapi@yahoo.gr (V.A.); louisegranlund29@gmail.com (L.G.); Robert.fredriksson@farmbio.uu.se (R.F.); sonchitab@gmail.com (S.B.); 3 Department of Neuroscience, Functional Pharmacology, Uppsala University, SE-751 23 Uppsala, Sweden; michel.edwar77@gmail.com; 4 Institute of Genetics and Animal Biotechnology of the Polish Academy of Sciences, ul. Postepu 36A, Jastrzębiec, PL05 552 Magdalenka, Poland

**Keywords:** solute carrier proteins, SNAT6, pre-synaptic terminal, excitatory neurons, glutamate–glutamine cycle, caveolin, Na^+^-dependent transporter, endocytosis

## Abstract

SLC38A6 (SNAT6) is the only known member of the SLC38 family that is expressed exclusively in the excitatory neurons of the brain. It has been described as an orphan transporter with an unknown substrate profile, therefore very little is known about SNAT6. In this study, we addressed the substrate specificity, mechanisms for internalization of SNAT6, and the regulatory role of SNAT6 with specific insights into the glutamate–glutamine cycle. We used tritium-labeled amino acids in order to demonstrate that SNAT6 is functioning as a glutamine and glutamate transporter. SNAT6 revealed seven predicted transmembrane segments in a homology model and was localized to caveolin rich sites at the plasma membrane. SNAT6 has high degree of specificity for glutamine and glutamate. Presence of these substrates enables formation of SNAT6-caveolin complexes that aids in sodium dependent trafficking of SNAT6 off the plasma membrane. To further understand its mode of action, several potential interacting partners of SNAT6 were identified using bioinformatics. Among them where CTP synthase 2 (CTPs2), phosphate activated glutaminase (Pag), and glutamate metabotropic receptor 2 (Grm2). Co-expression analysis, immunolabeling with co-localization analysis and proximity ligation assays of these three proteins with SNAT6 were performed to investigate possible interactions. SNAT6 can cycle between cytoplasm and plasma membrane depending on availability of substrates and interact with Pag, synaptophysin, CTPs2, and Grm2. Our data suggest a potential role of SNAT6 in glutamine uptake at the pre-synaptic terminal of excitatory neurons. We propose here a mechanistic model of SNAT6 trafficking that once internalized influences the glutamate–glutamine cycle in presence of its potential interacting partners.

## 1. Introduction

Among the membrane proteins that make up around 27% of all proteins in the human genome [1], the solute carriers are the second largest family with at least 430 members in humans [2]. The solute carriers, also known as SLCs, are responsible for the uptake and flow of several substances including amino acids, nucleotides, sugar, inorganic ions, and drugs across the cell membrane as well as excreting toxic products. These transporters, apart from being crucial for cell proliferation, growth and survival, are also pharmacologically indispensable as drug targets [3]. However, research on them is rather limited and there is a significant number of SLCs that is not characterized with their substrate profile, structure, localization or function [4].

Glutamate is one of the key regulators in the nervous system and imbalance in the glutamate–glutamine system may play a role in neuropsychiatric conditions, including schizophrenia, anxiety, bipolar disorder and to some extent, mood disorders [5,6]. Glutamate signaling is regulated at different levels by neurotransmitter release, re-uptake, and synthesis and hence can be affected by altered synaptic strength. The glutamate–glutamine cycle is the central and fundamental system that is involved in regulating all these aspects in order to fine tune and strictly control the strength of synaptic signaling at glutamatergic synapses to avoid the neurotoxic effects of glutamate [7], also known as excitotoxicity. This cycle is dependent on glutamine and glutamate transporters that are distributed among neurons and astrocytes in a tightly regulated manner [8]. There are 11 members in SLC38 family of glutamine transporters that control the glutamate–glutamine cycle in the brain. SLC38A6 (SNAT6) is one of the members of the family that has been recognized as an orphan transporter with unknown substrate profile [9]. In this study, we have illustrated that glutamine and glutamate are the preferred substrates of SNAT6. 

Only a few members of the SLC38 family have been identified as crucial players in the glutamate–glutamine cycle in the brain [10]. In the glutamate–glutamine cycle, glutamate can be taken up by the presynaptic glutamate transporter EAAT (Excitatory amino acid transporter) into neurons [11], but in contrast to most other classical neurotransmitters this contributes to only a small proportion of the total glutamate clearance. It is generally believed that the uptake into the glial cell constitutes the most important removal mechanism, a transport mediated by glutamate transporters GLAST and GLT-1. After uptake into the glial cells, glutamate is converted into glutamine, a non-excitotoxic amino acid, via glutamine synthetase. Then glutamine is transported out of the glial cell into the extracellular space, most likely via SNAT3 and SNAT5 [10,12]. From the extracellular space glutamine is taken up into the pre-synaptic glutamatergic neuron, originally thought to be mediated by the System A transporters SNAT1 and SNAT2, but more recent evidences have shown that these two transporters only contribute to a very minimal extent [13,14]. Hence, molecular identity of the neuronal part of this fundamental system in the brain is currently unknown. SNAT6 has been shown to be expressed exclusively on excitatory neurons in the brain [9]; thus making it the most promising candidate identified so far as being the long sought for neuronal glutamine uptake transporter. 

There is limited knowledge about the interacting partners of SNAT6 [9]. To understand the interacting proteins of SNAT6 we used a method called Algorithm for the Reconstruction of Accurate Cellular Networks (ARACNE). ARACNE promises in identifying direct transcriptional interactions in mammalian cellular networks. Exploring the network would enable identification of real physical interactions when gene network with information from sequence data is integrated with experimental data [15]. Microarray expression profiles, can be used to scale up the complexity of regulatory networks in mammalian cells, although general enough to address a large range of network deconvolution problems [16,17]. In this study, we exploited this approach to explain the data we acquired using microarray [18] to elucidate functional relationships that underlie cellular processes.

The substrate binding and regulation mechanisms of EAAT/EAAC family of proteins has remained elusive. Action of EAATs are localized to the plasma membrane which include dynamic regulation and intracellular trafficking. EAAT2, localizes to cholesterol-rich lipid raft microdomains where its intracellular trafficking between plasma membrane and endocytic compartment is regulated during synaptic transmission [19,20]. On the other hand, EAAC1 has been associated with Caveolin-1 aiding in trafficking of EAAC1 on and off the plasma membrane. These effects are associated with formation of EAAC1-caveolin complexes [11].

Caveolae are 50–100-nm plasma membrane structures expressed in adipocytes, endothelial cells, muscle cells, and fibroblasts in three different isoforms all of which are expressed in neurons. Caveolae have been associated with endocytosis [21]. We have addressed the structure of SNAT6, its substrate specificity, membrane localization, transport and internalization. We further studied how SNAT6 cycles between cytoplasm and plasma membrane in a caveolin dependent manner based on availability of substrates. This results in interactions with Pag, synaptophysin, CTPs2, and Grm2. Overall, the data suggest a mechanism of glutamine uptake via SNAT6.

## 2. Results

### 2.1. Structural Representation of SNAT6 Protein with TMS Prediction and 3D Modeling

**Figure 1:**
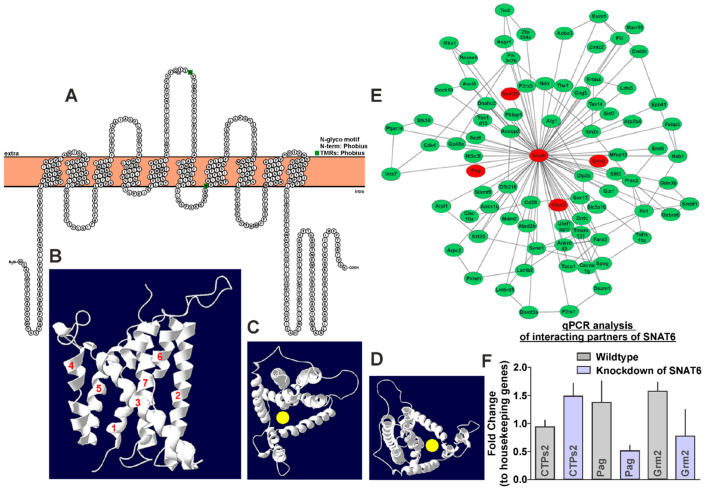
Predicted structure of SNAT6 and analysis of proteins influencing or being influenced by Scheme 6. (**A**)Topology prediction of SNAT6 is used to visualize the transmembrane segments using the software ‘Protter’ that enables interactive protein feature visualization and integration of experimental proteomic data. (**B**) Predicted 3D structure of SNAT6 with 7 transmembrane segments is obtained using the Swiss-Model, where a structurally known arginine-agmatine antiporter protein is used as template. (**C**,**D**) The top-views of SNAT6 exhibit a possible substrate pore (marked in yellow) for transport. (**E**) ARACNE generated network of proteins influencing or being influenced by SNAT6. SNAT6 and four other proteins namely CTP Synthase 2 (CTPs2), Phosphate activated glutaminase (Pag), Glutamate metabotropic receptor 2 (Grm2) and Snat10 are marked red and were chosen for further analysis. (**F**) qPCR analysis revealed upregulation of CTPs2, and downregulation of Pag and Grm2 in the immortalized embryonic mouse hypothalamic cell line where SNAT6 was knocked down using specific siRNA. The qPCR experiments were performed 2–3 times on separate batches of cell line and the data were normalized for cell number differences between untreated control cells seeded on the same set of plates. An unpaired t-test with 95% confidence interval was performed showing significant downregulation for Pag and Grm2 (*p* < 0.001).

We predicted the structural appearance of SNAT6 to see if it shared structural characteristics with the known SLCs. We used transmembrane segment (TMS) prediction software ‘Protter’ [22] that enables interactive protein feature visualization and integration of experimental proteomic data, to obtain the TMS characteristics of SNAT6. It was predicted to have eleven TMS domains with a relatively long intra-cellular N-terminus and a shorter extra-cellular C-terminus (Figure 1A). However, the homology model of SNAT6 that is built using SWISS-MODEL [23], displayed only seven possible TMS (Figure 1B). The protein sequence was aligned against a structurally known arginine–agmatine antiporter protein template [23,24,25], where the SNAT6 model had global model quality estimation (GMQE) value of 0.27. GMQE scale range from 0 to 1, where higher numbers indicate a better model. The tertiary structures (Figure 1C,D) from top view indicated formation of a pore, through which a substrate possibly could be transported through SNAT6. 

### 2.2. Several Genes Relevant to Glutamate–Glutamine Cycle Are Predicted to Interact with SNAT6

Several genes were found to be influencing or being influenced by SNAT6 (Figure 1E, Table S1) using ARACNE. The highest-ranking genes were *CTP Synthase 2* (also annotated as *CTPs2*), *Phosphate activated glutaminase* (also annotated as *Pag*), and *Glutamate metabotropic receptor 2* (also annotated as *Grm2*). Interestingly, SLC38A10 (also annotated as Snat10) from the same family as SNAT6, that has recently been characterized as a transporter for glutamine, glutamate and aspartate [26], was found to be interacting with SNAT6.

### 2.3. siRNA-Induced Knockdown of SNAT6 Shows Upregulation or Downregulation of Predicted Interacting Genes

In order to record any change in gene expression of these potential interacting partners of SNAT6 in its absence, specific siRNA was used to knock down SNAT6 expression in an immortalized mouse hypothalamic cell line. Real time qPCR on cDNA (extracted separately from wild type and siRNA treated cells) revealed that CTPs2, Pag, and Grm2 had the highest relative fold change when SNAT6 expression was knocked down in the cell, compared to some other proteins revealed in the analysis. CTPs2 was upregulated in the siRNA treated cells whereas Pag and Grm2 were downregulated in comparison to the wild type untreated cells (Figure 1F). We did not observe any change in SLC38A10 in these experiments.

### 2.4. CTPs2 Shows Similar Histological Profile as SNAT6

**Figure 2:**
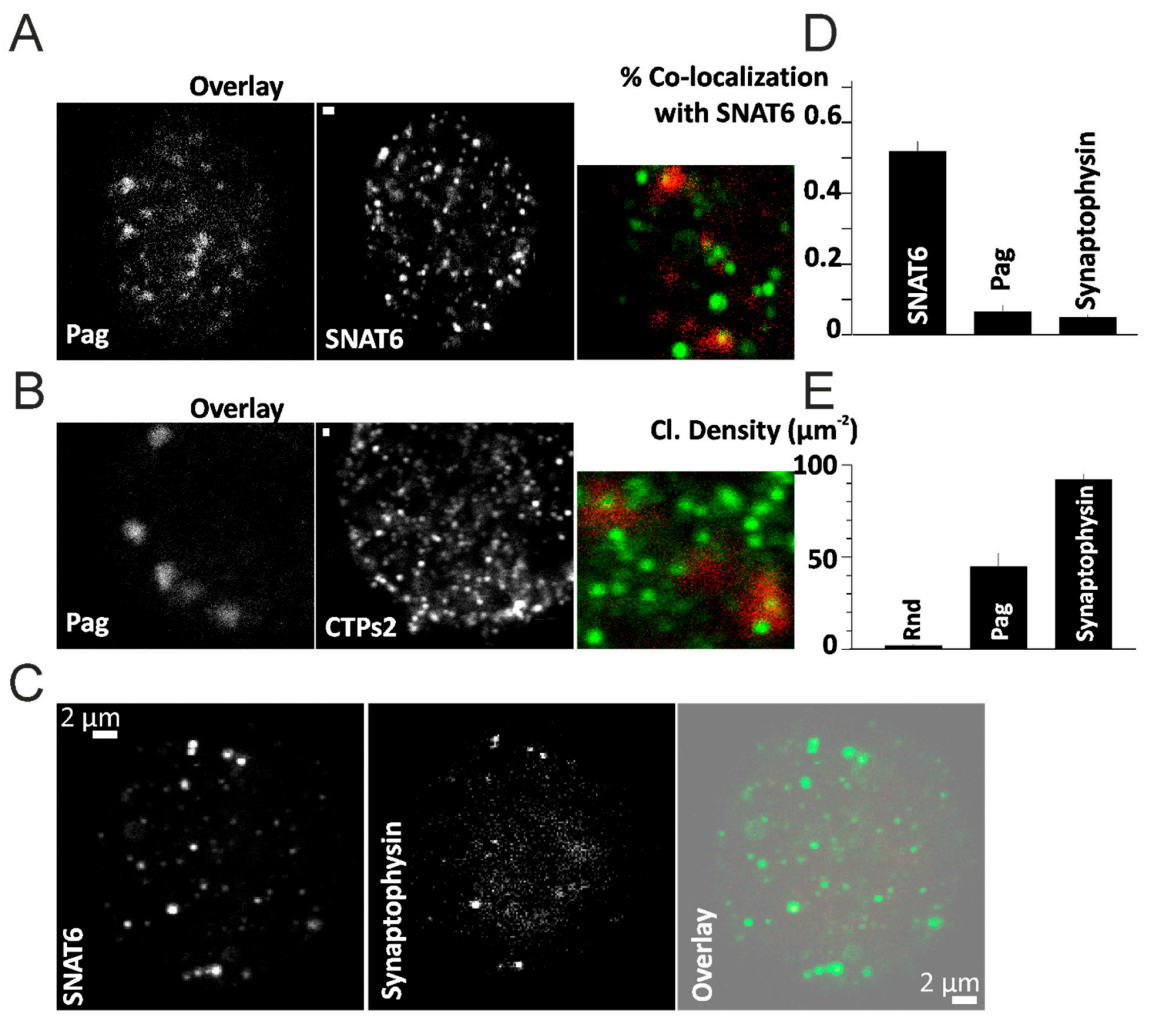
Localization of Pag and Synaptophysin. (**A**) Immunostained images of Pag and SNAT6 with an overlay image in the inset. (**B**) Immunostained images of Pag and CTPs2 with an overlay image in the inset. Scale bar 2 µM in (**A**,**B**). (**C**) Immunostained images of SNAT6 and Synaptophysin with an overlay image on the right. Scale bar 2 µM in (**C**). (**D**) Co-localization of SNAT6 with Pag and Synaptophysin measured as described in the methods. Random (Rnd) regions selected were used as control. Both Pag and Synaptophysin co-localized with SNAT6 at a significantly (*p* < 0.001) higher percentage compared to Rnd regions (**E**) Density of clusters of SNAT6, Pag and Synaptophysin was assessed as described in the methods. Note of density of SNAT6 which is significantly (*p* < 0.001) higher than Pag or Synaptophysin. All the experiments in the figure were repeated for 2–3 times each with each of the bar graph representing more than 15 cells obtained from separate experiments.

We have previously shown that SNAT6 co-localizes with Pag (phosphate-activated glutaminase) (Bagchi et al., 2014). This was further confirmed with immunostaining. Here we observed the endogenous proteins localization of Pag that co-localized with SNAT6 (Figure 2A). A similar co-localization was observed with a synaptic marker for presynaptic vesicle glycoprotein-synaptophysin. The synaptophysin co-localization with SNAT6 was much higher than its co-localization with Pag (Figure 2C). The density of puncta of SNAT6 in the cell was much higher than the density of Pag or Synaptophysin (Figure 2D,E). 

**Figure 3:**
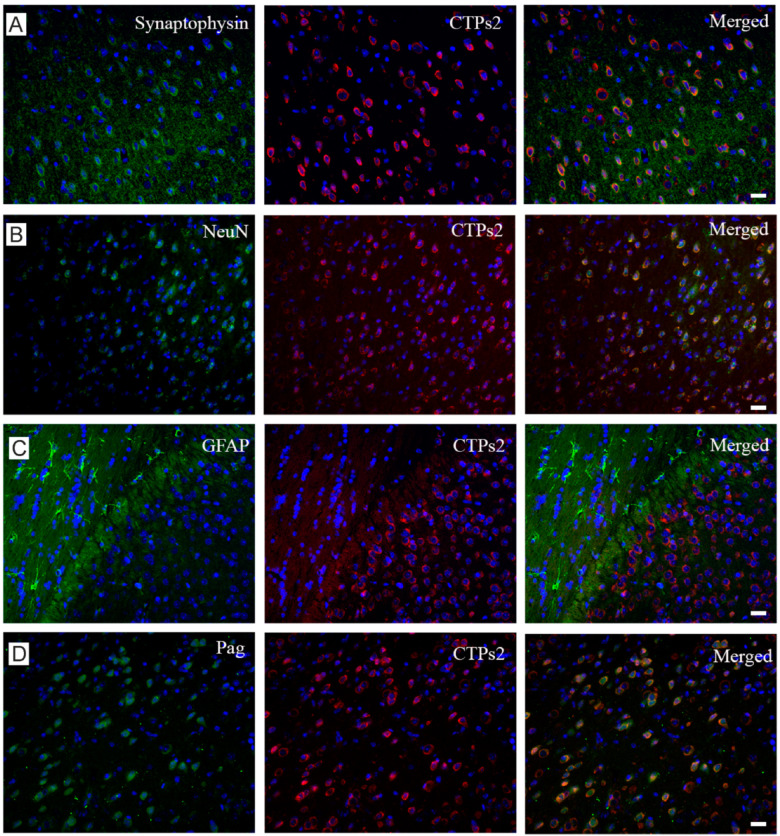

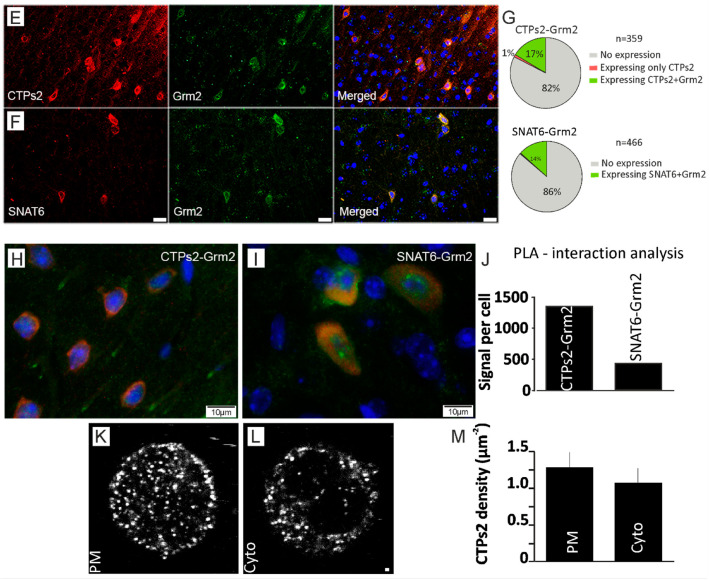
Downstream role of Pag, CTPs2, Grm2 and NeuN in glutamate-glutamine cycle. (**A**) This horizontal panel shows staining with synaptophysin, CTPs2 and their merged image from left to right. Scale bar 10 µM (**B**) Horizontal panel shows staining with NeuN, CTPs2 and their merged image from left to right. Scale bar 10 µM (**C**) Horizontal panel shows staining with GFAP, CTPs2 and their merged image from left to right. Scale bar 10 µM (**D**) Horizontal panel shows staining with Pag, CTPs2 and their merged image from left to right. All micrographs have nucleus stained in blue with DAPI. The first vertical column exhibits different markers in green (Alexa fluor 488), whereas the middle column illustrates CTPs2 staining in red (Alexa fluor 594) in all four micrographs. The last column shows merged images of first two columns. Scale bar 10 µM (**E**) Horizontal panel shows staining with CTPs2, Grm2 and their merged image from left to right. (**F**) Horizontal panel shows staining with SNAT6, Grm2 and their merged image from left to right. All micrographs have nucleus stained in blue with DAPI. The first vertical column exhibits CTPs2 (top) and SNAT6 (bottom) in red (Alexa fluor 594), whereas the middle column illustrates Grm2 staining in green (Alexa fluor 488) in both micrographs. The last column shows merged images of first two columns. Scale bar 10 µM for E and F (**G**) Pie diagrams to illustrate that the protein pairs were not found in all the cells from mouse brain tissue, but they always co-stained the cells they were detected in. For co-staining of CTPs2 and Grm2, 359 cells were manually counted to reveal no expression in 82% of cells, only CTPs2 expression in 0.84% (rounded up to 1% in Figure 3) of cells, only Grm2 expression in 0% of cells and co-expression in 17% of cells. For co-staining of SNAT6 and Grm2, 466 cells were manually counted to reveal no expression in 86% of cells, only SNAT6 expression in 0.43% (rounded up to 0% in Figure 3) of cells, only Grm2 expression in 0% of cells and co-expression in 14% of cells. (**H**) Double immune-staining with CTPs2 in red (Alexa fluor 594), Grm2 in green (Alexa fluor 488) and nucleus in blue (DAPI). (**I**) Double immune-staining with SNAT6 in red (Alexa fluor 594), Grm2 in green (Alexa fluor 488) and nucleus in blue (DAPI). (**J**) Proximity ligation analysis (PLA) shows 1343.33 signals per cell for protein pair CTPs2 and Grm2 and 428.5 for SNAT6 and Grm2. The experiments were repeated multiple times in the same manner. (**K**,**L**) Immunostained images of CTPs2 at the plasma membrane (PM) and cytosol (cyto) of the cell. Scale bar 1 µM (**M**) Density of CTPs2 clusters was assessed using plugin called “find maxima” described in the methods. These experiments were repeated twice with at least 13 cells per time.

We wanted to further visualize the expression pattern of CTPs2 in mouse brain sections to compare with that of SNAT6. Co-immuno labeling of CTPs2 with different neural markers exhibited its expression in excitatory neurons and at synapses (seen from merged image at the extreme right, with nuclear staining in blue with DAPI), but not in the glial cells (Figure 3A–D). This is a very similar expression profile as that of SNAT6 (Bagchi et al., 2014). CTPs2 co-localized with synaptophysin (Figure 3A horizontal panel) (Wiedenmann and Franke, 1985), revealing its presence in the synapse. CTPs2 also co-localized with NeuN and Pag which are markers for neuron-specific DNA-binding protein (Mullen et al., 1992) and for an enzyme which generates glutamate and ammonia from glutamine in excitatory neurons (Manns et al., 2001; Van Der Gucht et al., 2003) respectively (Figure 2B and Figure 3B,D horizontal panels). These results confirm expression of CTPs2 in excitatory neurons. However, no co-localization was observed between CTPs2 and GFAP that is a marker for glial fibrillary acidic protein on astrocytes (Reeves et al., 1989) (Figure 3C horizontal panel), concluding the absence of CTPs2 in the astrocytes.

### 2.5. Grm2 Is Co-Expressed with both SNAT6 and CTPs2

Because SNAT6 and CTPs2 both revealed similar expression pattern, we studied co-localization of Grm2 with SNAT6 and CTPs2 with immunohistochemistry to understand its distribution in cells and illustrate relative expression of these three proteins (Figure 3E,F horizontal panels) with a merged image at the far right. The nucleus is stained in blue with DAPI in all the micrographs. Clearly, they are not expressed in all cells, but are always co-expressed. To quantify this finding, we manually calculated expression of CTPs2 and Grm2 in 359 cells and that of SNAT6 and Grm2 in 466 cells. The pie diagrams (Figure 3G) show that in both cases, the majority of cells either expresses both of the proteins together or none at all. 

Interestingly at subcellular level, our staining showed complete overlap between Grm2 and CTPs2 whereas the green staining of Grm2 and the red staining of SNAT6 were rather complementary to each other (Figure 3H,I). This was in spite of CTPs2 localized equally in the plasma membrane apart from its cytosolic localization (Figure 3K–M). To explore this expression pattern further, we used proximity ligation assays (PLA) with the same combination of proteins. Upon quantification of PLA signals per cell, Grm2 and CTPs2 seem to be in closer proximity than Grm2 and SNAT6 (Figure 3J). There were more PLA signals recorded for Grm2 and CTPs2 compared to those for Grm2 and SNAT6, which complements the pattern we observed in the immunohistochemistry staining.

### 2.6. Glutamine and Glutamate Are Preferred Substrate for SNAT6

**Figure 4:**
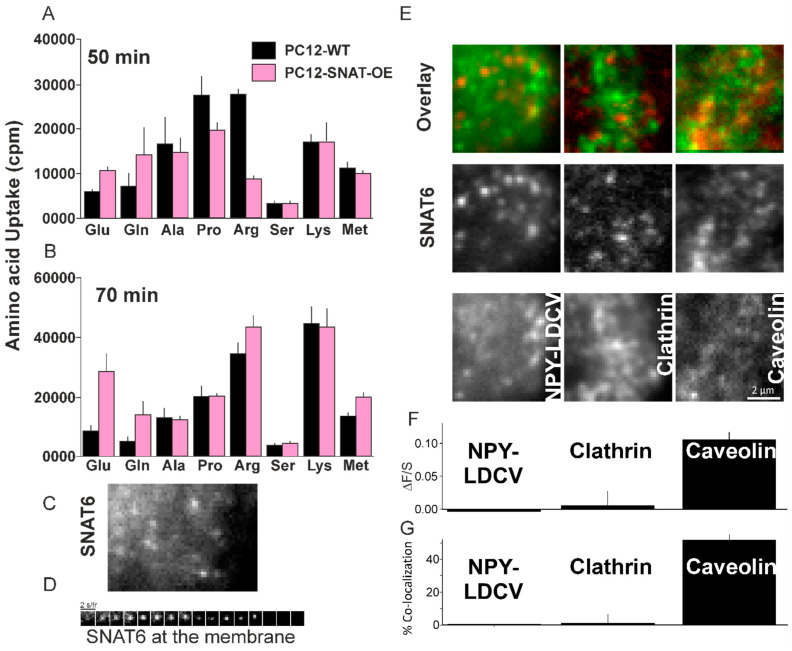
Role of SNAT6 in amino acid uptake and internalization. (**A**) Uptake in counts per minute (cpm) for 50 min by wildtype PC12 cells and PC12 cells overexpressing SNAT6 are plotted. (**B**) Uptake in counts per minute (cpm) for 70 min by wildtype PC12 cells and PC12 cells overexpressing SNAT6 are plotted. 3H-labeled Glutamine, glutamate, alanine, proline, arginine, serine, lysine and methionine are used for both time points. All measurements were performed in duplicates for multiple times and mean value is shown. Significantly higher uptake (*p* < 0.001) is shown for the amino acids - glutamine and glutamate in PC12-SNAT6-OE cells compared to PC12-wt cells after 50 mins. (**C**) A TIRF microscopy image showing cells overexpressing SNAT6-EGFP at the plasma membrane (**D**) Time series strip showing disappearance of SNAT6-EGFP from the plasma membrane in presence of glutamine. (**E**) Images of a cell overexpressing SNAT6-EGFP in red with NPY-mCherry or Clathrin-mCherry or Caveolin-mCherry in green channels respectively with a corresponding image in the overlay. (**F**) Quantification of binding to the SNAT6 site (ΔF/S) for NPY, clathrin and Caveolin respectively (described in methods). Localization of SNAT with Caveolin was significantly higher compared to NPY or clathrin (*p* < 0.001). (**G**) Co-localization from the images similar to E were analyzed (described in methods). SNAT co-localization with caveolin was significantly higher than its co-localization with NPY of clathrin (*p* < 0.005). All the TIRF imaging experiments were repeated at least 3 times with at least 8 cells per time.

In order to reveal a substrate profile for SNAT6, PC12 wildtype cells and PC12 cells overexpressing SNAT6 were used. These two cell-lines were incubated for 50 min with a series of 3H-labeled amino acids to estimate steady state levels of amino acids. Higher uptake of glutamine and glutamate was observed in the overexpressing cells compared to the wildtype cells (Figure 4A) although, various amino acids including alanine, proline, arginine, serine, lysine, and methionine were used for this experiment. This confirms that glutamate and glutamine are the preferred amino acids transported by SNAT6 during first 50 min and even after 70 min (Figure 4B).

### 2.7. SNAT6 Localizes with Caveolin1

In order to understand the membrane distribution of SNAT6 we expressed SNAT6 tagged to GFP in PC12 cells. SNAT6 had a punctate distribution (Figure 4C) and like other members of the family [11] SNAT6 also constitutively cycles on and off the plasma membrane (Figure 4D). We tested how this recycling affects delivery and endocytosis of SNAT6. When co-expressed with secretory granules marker NPY-mCherry, SNAT6 both had punctate distribution (Figure 4E) but very low degree of co-localization measured by ΔF/S (Figure 4F, see methods). A similar low degree of association was observed when SNAT6 was co-expressed with Clathrin-mCherry. When SNAT6 was co-expressed with Caveolin1-mCherry there was a high degree of association. A 10-fold increase in ΔF/S was significantly higher than seen for clathrin. The association of labeled caveolin, clathrin, and NPY was independently assessed by measuring the overlaid distribution of both populations of puncta (as described in the methods) expressed as percentage. The results (Figure 4G) were similar to the method described in Figure 4F. We conclude that SNAT6 recycles at the plasma membrane and has a very high degree of association with caveolin1.

### 2.8. SNAT6 Associated Caveolin Complexes Internalize in Response to Glutamine and Glutamate

**Figure 5:**
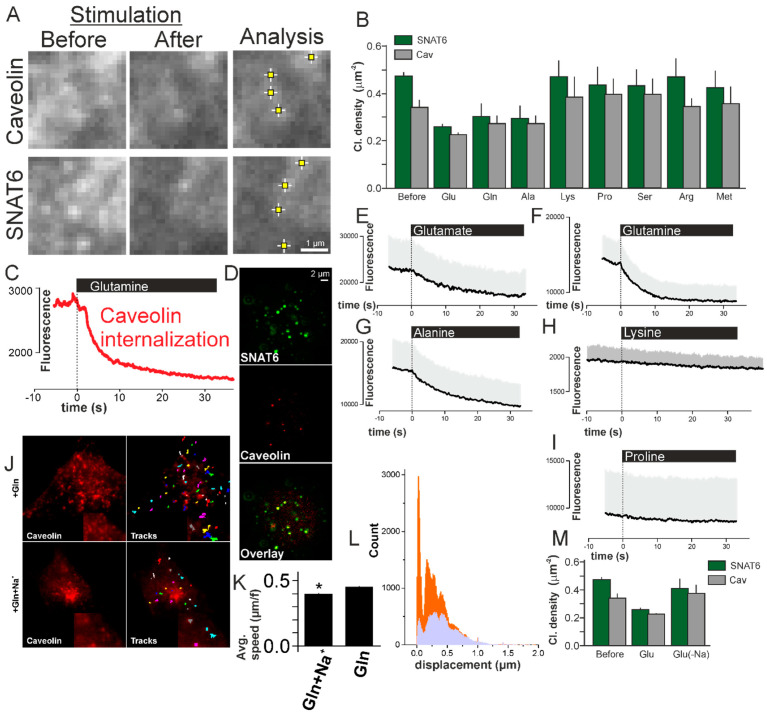
SNAT6 associated caveolin complexes internalize in response to glutamine and glutamate in a Na^+^ dependent manner. (**A**) Images of cells expressing SNAT6-EGFP and Caveolin1-mCherry under TIRF microscopy before and after stimulation with glutamine. An ImageJ plugin (described in methods) detecting the SNAT6 and caveolin clusters. (**B**) Density of SNAT6 (green) and Caveolin (grey) clusters quantified using the method above before and after stimulations with different amino acids specified in the figure (density of clusters was significantly decreased under Glu, Gln, and Ala, *p* < 0.001). (**C**) Trace showing fluorescence of the entire single cell for caveolin. Note that the stimulation with glutamine was initiated at time zero. (**D**) Images from antibody labeled SNAT6 and caveolin localizing together (44 ± 7% in six cells derived from two independent experiments). (**E**) Similar to C but showing average fluorescence for 10 cells from three different transfections under glutamate stimulation. (**F**–**I**) Similar to E for cells stimulated with glutamine (11 cells), alanine (13 cells), lysine (12 cells), and proline (9 cells). All the experiments were performed from at least three different transfections. (**J**) Images showing caveolin-mCherry expressing cells with and without Na^+^ present in the buffer when stimulated with glutamine. Tracks obtained from Metamorph tracking (described in methods) during the entire time sequence. zoomed tracks and images in the inset (**K**) Average speed for the tracks in (**J**) in presence and absence of Na^+^ in the buffer under glutamine stimulation (* *p* < 0.05). (**L**) Histogram of 16–60 tracks from 9 different cells per condition similar to the cell shown in (**I**). Displacement of caveolin in presence (lavender) and absence (orange) of Na^+^ in the buffer under glutamine stimulation. (**M**) Density of SNAT6 (green) and Caveolin (grey) clusters quantified as in B before and after stimulations with glutamine in presence and absence of Na^+^ in the buffer.

SNAT6 association with caveolin at the plasma membrane was confirmed by immunostaining (Figure 5D) and the functionality of the transporter was evaluated in presence of SNAT6 substrates (tested previously in Figure 4A,B) that include alanine, proline, arginine, serine, lysine, and methionine apart from glutamate and glutamine. Live cells were incubated for 2 min in presence of each of the amino acids and images acquired before and after incubation (Figure 5A). To further substantiate the findings, the number of puncta forming clusters from SNAT6 and caveolin were counted automatically using an ImageJ function named “find maxima” [27]. The density of SNAT6 and Caveolin decreased after incubation with glutamate, glutamine, and alanine with none of other amino acids showing any effect on SNAT6 and caveolin (Figure 5B). 

To evaluate this effect in real-time cells expressing labeled SNAT6 and caveolin were imaged for 1 min. Stimulation with glutamate, glutamine, alanine, lysine and proline respectively was triggered after 10s using a patch-pipette placed over the cell. When cells were stimulated with glutamine the fluorescence of labeled caveolin decreased by half within 30 secs (Figure 5C). A similar effect was observed in a more robust manner with at least 9 cells per experiment with glutamate and alanine but not with lysine and proline (Figure 5E–I). With this we can conclude that SNAT6 associates with caveolin in the plasma membrane (Figure 5D) to form complexes that aid in internalization of them in response to SNAT6 substrates such as glutamine and glutamate.

### 2.9. SNAT6 Associated Caveolin Internalization Is Dependent on Availability of Na^+^

SNAT6 functions in presence of Na^+^ [28]. We tested the functioning of SNAT6 and how it affects Caveolin dependent internalization in absence of Na^+^. Cells overexpressing labeled caveolin and SNAT6 were stimulated as in previous experiments in presence and absence of Na^+^ in the extracellular buffer. The internalization here was monitored in real-time by tracking the caveolin rich sites using a Metamorph tracking plugin (Figure 5J, zoomed tracks in the inset). A histogram plotted from the tracks obtained showed fewer minimum displacement tracks in presence of the Na^+^ compared to in absence of Na^+^ (Figure 5L). The average speed obtained from these tracks was slower in presence of the Na^+^ compared to absence of Na^+^ (Figure 5K). Lower number of tracks combined with lower speed and fewer clusters (Figure 5B) show that there are fewer SNAT6–caveolin complexes due to efficient internalization in presence of substrate. This could be a cellular response to high intracellular concentrations of amino acids, to reduce further uptake. Blocking this internalization in absence of Na^+^ results in more SNAT6–caveolin complexes unable to internalize at the plasma membrane resulting in more tracks. To further confirm the effect of Na^+^_,_ we measured the density of SNAT6 and caveolin clusters at the plasma membrane in presence and absence of Na^+^. The number of clusters in absence of Na^+^ was similar to the number of clusters without substrate stimulation whereas in presence of Na^+^ the complexes had internalized (Figure 5M). Therefore, SNAT6 functions in Na^+^ dependent manner to associate with caveolin and internalize.

### 2.10. SNAT6 Alocalization and Downstream Signaling

**Figure 6:**
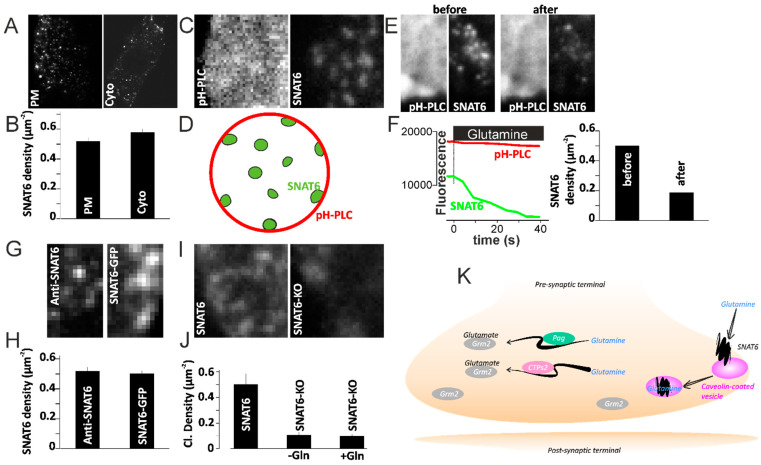
SNAT6 downstream signalling. (**A**) Immunostained images of SNAT6 at the plasma membrane (PM) and cytosol (cyto) of the cell. (**B**) Density of SNAT6 clusters was assessed using plugin called “find maxima” described in the methods. (**C**) Cells co-transfected using PtdIns(4,5)P_2_ labeled with PH domains of PLCδ1-mRFP (pH-PLC) and SNAT6 labeled with GFP images obtained using TIRF microscopy. (**D**) Cartoon showing SNAT6 distribution in the cell and PIP_2_ at the plasma membrane. (**E**) Images as in C for cells before stimulating with glutamine and after stimulating with glutamine. (**F**) Trace showing fluorescence of the entire single cell for SNAT6 (green) and PIP_2_ marker pH-PLC (red). Note that the stimulation with glutamine was initiated at time zero. The density of SNAT6 clusters before and after stimulation is shown on the right. (**G**) Images showing SNAT6 immunostained using a specific antibody and SNAT6 tagged to GFP. (**H**) Density of SNAT6 from the images similar to displayed in E analyzed using “find maxima” as described in the methods. (**I**) Images from SNAT6 immunolabeled cells fixed after siRNA mediated knockdown of SNAT6 or untransfected control cells. (**J**) Density of immunostained SNAT6 from the untrasfected cells or cells undergone siRNA mediated knockdown of SNAT6 fixed with or without prior Gln stimulation analyzed using “find maxima” as described in the methods. Note SNAT6-KO cells have significantly fewer clusters (*p* < 0.001). All experiments were repeated two or three times independently and each bar graphs represents 12–50 cells each. (**K**) A proposed model illustrating how SNAT6 might be regulating the glutamate–glutamine cycle: SNAT6 transports in glutamine from astrocytes to the pre-synaptic terminal of excitatory neurons. SNAT6–caveolin complexes internalize upon binding to glutamine. Two enzymes Pag and CTPs2 then convert the glutamine to glutamate via two separate pathways. The glutamate produced then binds to the glutamate-receptor Grm2 prior to its release.

The above results suggest that the substrate specific binding of SNAT6 and its internalization with caveolin triggers further downstream signaling. For constant availability of SNAT6 its availability at any given point should not be limited at the plasma membrane. We therefore assessed the percentage of endogenous SNAT6 localized to plasma membrane compared to the cytosolic compartment. Endogenous SNAT6 showed a punctate form of distribution similar to EGFP tagged SNAT6. The distribution at the plasma membrane was very similar in the endogenous and overexpressed SNAT6 (Figure 6E–F). The plasma membrane localization in these experiments were confirmed using and a homogenously labeled PIP_2_ marker tagged to RFP [29,30] at the plasma membrane where SNAT6 puncta localized when visualized under the TIRF-field (Figure 6C–D). When these cells were stimulated as described in Figure 5C with glutamine, the fluorescence of homogenously labeled PIP_2_ marker tagged to RFP remained homogenous and similar to the fluorescence seen before stimulation (Figure 6E). The cluster density and the fluorescence intensity of SNAT6 decreased dramatically and halved (Figure 6F, left panel-green trace and right panel) while the PIP_2_ remained unaffected (Figure 6F, left panel) confirming that the SNAT6 internalization is independent of membrane ruffling or any structural changes in the plasma membrane. Endogenous SNAT6 distributed in the cytosol similar to its distribution at the plasma membrane (Figure 6G–H). The fraction of SNAT6 in the cytosol compared to the plasma membrane was similar (Figure 6A,B). The functionality of plasma membrane localized SNAT6 was assessed in SNAT6-knockdown cells where the number of SNAT6 puncta were significantly fewer at the plasma membrane. The small number of plasma membrane localized SNAT6 puncta in the knockdown cells remained even with or without addition of Gln prior to fixation (described in methods) confirming that they were not functional (Figure 6I,J). Therefore, substrate-specific binding of SNAT6 and internalization is crucial for downstream signaling.

## 3. Discussion

In our homology model, we could identify seven transmembrane domains of SNAT6. We have used uptake assays with labeled amino acids and real time live cell imaging to demonstrate that SNAT6 is a glutamine and glutamate specific transporter.

SNAT6 could play a role in fine tuning the balance of glutamine and glutamate at the synapses of excitatory neurons where it is exclusively expressed (Bagchi et al., 2014). Here, we identified several interesting genes—such as CTPs2, Pag, and Grm2—that seem to be affected in cells where SNAT6 protein was knocked down leading to decreased amino acid transport (Figure 1F, Figure 2D,J and Figure 6K). We have shown that Pag or Phosphate activated glutaminase, which is exclusively expressed in excitatory neurons, is co-localized with SNAT6 (Figure 2B–E) (Bagchi et al., 2014). In shortage of SNAT6, less glutamine is transported into the cell and hence fewer enzymes (Pag) are required to convert glutamine to glutamate and ammonia, leading to downregulation of Pag. The rate limiting enzyme CTP synthase requires sufficient glutamine in order to catalyze formation of CTP from UTP by releasing phosphate with the concomitant deamination of glutamine to glutamate. In absence of sufficient levels of glutamine, CTPs2 is upregulated in an attempt to increase the production of glutamate, probably as a compensation mechanism. The glutamate now produced from glutamine binds to metabotropic glutamate receptor, Grm2 that is also expressed in the presynaptic ends of excitatory neurons [31]. The metabotropic glutamate receptors (also known as mGluRs) are a family of G-protein-coupled receptors that bind glutamate and transmit signals to the intracellular signaling partners (Niswender and Conn, 2010). In absence of SNAT6, the level of glutamine reduces and hence the level of glutamate is also decreased in the cells leading to a negative feedback effect that causes downregulation of Grm2 to compensate for limited availability of glutamate. However, it has to be taken in to account that the protein-to-mRNA ratio in steady state varies in a direction that lessens the change in protein levels [32], which was not assessed in our study.

Originally, caveolae were described as invaginations at the plasma membrane involved in endocytosis. Since then, caveolae have been implicated in the internalization of a number of diverse molecules [21]. Disrupting the raft containing caveolae results in decreased glutamate and serotonin transporter-mediated uptake [19,33]. Caveolin also regulate trafficking of plasma membrane transporters. Glucose transporter trafficking is caveolin dependent and lack of caveolae results in reduced glucose uptake [34]. EAAC1 has been associated with Caveolin-1 aiding in its trafficking on and off the plasma membrane. These effects are associated with formation of EAAC1-caveolin complexes [11] for endocytosis in glutamate metabolism [35]. Here we identified a mechanism for SNAT6 internalization similar to the mechanisms seen for the family member EAAC1. SNAT6 forms complexes with caveolin in presence of SNAT6 substrates to internalize and this interaction is sodium dependent. Taking sodium out almost acts as an inhibitor for the association of SNAT6 functioning. This is in line with observation of other members of the family where they acts as secondary active transporters energized by the electrochemical gradient of sodium [36].

Our data also illustrates SNAT6 as a glutamine/glutamate transporter that contributes to maintain balance between glutamine and glutamate, exclusively at the pre-synaptic terminal of the excitatory neurons. We propose a simplified model (Model Figure 6K) that demonstrates the probable role of SNAT6 in the glutamate–glutamine cycle at the pre-synaptic terminal of excitatory neurons. SNAT6 is localized to caveolin rich areas at the plasma membrane. In presence of SNAT6 specific substrates such as glutamine and glutamate, SNAT6 internalizes in a caveolin dependent manner, possibly in response to high intracellular amino acid concentrations. Evidence from SNAT6 localization at the plasma membrane and cytosol compartments in cultured cells (Figure 6A,B) shows how change in localization of SNAT6 acts as a trigger for amino acid transport. However, there is a difference in appearance of SNAT6 in the immunofluorescence in the cells versus the tissue probably due to (1) different fixation protocols, which tends to destroy or alter cellular sub-structures as described previously [37,38], (2) a consequence of different nutrient or neurotransmitter availability in cells versus tissue. Of course, the observed difference could also reflect that the localization of this protein in cultured cell is different from its localization in vivo.

The amino acid is transported in the form of glutamine and is converted into glutamate and ammonia by the enzyme Pag. Another enzyme, CTPs2, is also converting glutamine to glutamate. Since glutamate is crucial for the release of neurotransmitters as well as causes neurotoxicity, it is understandable that this process is very firmly regulated. In absence of sufficient SNAT6, Pag is downregulated and CTPs2 is upregulated in order to balance the level of glutamate due to reduced transport of glutamine. The glutamate produced then binds to the glutamate-receptor Grm2 prior to its release. This model contributes to trafficking and functioning of SNAT6 and related proteins, as well as their possible involvement in the glutamate–glutamine cycle. Here we have shown the mechanisms behind trafficking of SLC38A6 in the glutamate–glutamine cycle in neuronal cells. The trafficking mechanisms could be functioning in other cell types where SLC38A6 expression is found which includes pancreas [39], cartilage [40], circulatory tissue [41], etc. 

## 4. Methods

### 4.1. Sequence and Homology Modeling

The transmembrane (TM) prediction software ‘Protter’ [22] was used for interactive protein feature visualization and integration with experimental proteomic data to obtain the proteins topologies. The fully automated homology SWISS-MODEL program [23] was used to build 3D models, where different structurally-known transporters were used as templates. The protein sequence of SNAT6 was aligned against the best-fit arginine–agmatine antiporter protein template [23,24,25], where the SNAT6 model had global model quality estimation (GMQE) value of 0.27. GMQE scale range from 0 to 1, where higher numbers indicate a better model. Manual inspections of the alignments were performed to enhance credibility of the models. Tertiary structures were finalized using Swiss-Pdb Viewer [42] and Adobe Photoshop CS6.

### 4.2. Microarray Data and ARACNE Analysis for SNAT6

In this study, ARACNE was used in order to explore the data acquired by using previously obtained microarray analysis [18] that is publicly available in NCBI-GEO database with accession number GSE61402. In the microarray study, 28,270 genes from immortalized mouse hypothalamic cell line N25/2 that were systematically deprived of amino acids for 1, 2, 3, 5, and 16 h were analyzed. The raw data was normalized using the robust multi-array average (RMA) method [43,44] and then further standardized to remove batch effect utilizing ComBat [45]. Finally, network inference was performed using mutual information of genes expression in ARACNE as described in Margolin et al. [16]. Network graphs were generated using Cytoscape [46]. The complete microarray data was loaded to ARACNE where mutual information index was calculated in reference to 2500 markers to generate the network. Only interactions with highest mutual information index were accepted for further investigation.

### 4.3. Cell Cultures and Cell Lines

The immortalized embryonic mouse hypothalamic cell line N25/2 (mHypoE-N25/2, CellutionsBiosystems Inc., Toronto, ON, Canada) was cultured in Dulbecco’s modified Eagle medium (DMEM [+] 4.5g/L D-Glucose, [+] L-Glutamine, [+] Pyruvate) from Gibco (Life technologies, Gaithersburg, MD, USA) supplemented with 50 mL fetal bovine serum (FBS) (Gibco, Life Technologies, Gaithersburg, MD, USA), 5 mL Penicillin-Streptomycin (Pen-Strep) and 5 mL amphotericin B (Gibco, Life Technologies, Gaithersburg, MD, USA). 

The immortalized rat adrenal gland cell line PC-12 Adh (CRL-1721.1, ATCC, Manassas, VA, USA) was cultured in ATCC-formulated complete growth media F-12K (catalog no. 30-2004) supplemented with 12.5 mL fetal bovine serum (FBS) (Gibco, Life Technologies, Gaithersburg, MD, USA), 75 mL horse serum (Gibco, Life Technologies, Gaithersburg, MD, USA), 5 mL Penicillin-Streptomycin (Pen-Strep) (Gibco, Life Technologies, Gaithersburg, MD, USA) and 5 mL amphotericin B (Gibco, Life Technologies, Gaithersburg, MD, USA). 

A stable cell line was created by antibiotic (Geneticin G418; Gibco, Life Technologies) selection of PC-12 Adh cells where a TrueORF vector (pCMV6-entry from OriGene, Rockville, MD, USA) overexpressing *SLC38A6* was transfected. Same media as that for PC-12 Adh wildtype cells were used with the exception of addition of Geneticin (Gibco, Life Technologies, Gaithersburg, MD, USA) for each culture. 

All the cells were incubated at 37 °C with 5% CO_2_. Cells were seeded on glass coverslips (coated with 10 μg/mL poly-l-lysine) for 40 h prior to immuno-staining.

Transient transfections were performed on 25-mm poly-l-lysine–coated coverslips in 100 µL OptiMEM^®^ (Life Technologies, Gaithersburg, MD, USA) using 0.5 µL Lipofectamine® 2000 (Life Technologies, Gaithersburg, MD, USA), 0.2–0.6 µg plasmid DNA, and 150,000 cells. The reaction was terminated after 3–5 h, and imaging was performed 24–30 h after transfection.

### 4.4. Knockdown of SNAT6

The cells from the immortalized mouse hypothalamic cell line were seeded in 6-well plates up to 60–80% confluency. Then 9 μL Lipofectamine^®^ RNAiMAX (Life Technologies, Gaithersburg, MD, USA) was diluted in 150 μL Opti-MEM^®^ Medium (Life Technologies) and 20 pmol SNAT6 siRNA (Ambion^®,^ Life Technologies, Gaithersburg, MD, USA) was diluted in 150 μL Opti-MEM^®^ Medium separately. The diluted siRNA was then mixed with the diluted Lipofectamine^®^ RNAiMAX Reagent in a 1:1 ratio and was incubated for 5 min at room temperature. From the siRNA-lipid complex, 250 μL was added to the cells which were incubated for 1–4 days at 37 °C with 5% CO_2_. Mock transfected cells were treated only with Lipofectamine^®^ RNAiMAX without siRNA and hence were used as a negative control. For microscopy experiments PC12 cells seeded on glass coverslips were transfected with SNAT6 siRNA as described above. The cells were fixed using 4% para-formaldehyde after 24 h. Prior to fixation cells were treated with Gln or a control solution without any amino acid for 10 min. Non-transfected cells were treated as external control. All these experiments were performed at least twice.

### 4.5. Quantitative Real-Time PCR (qPCR) and Data Analysis 

Both wildtype and siRNA treated cells, were harvested and total RNA was extracted using miRNeasy Micro Kit (Qiagen, Hilden, Germany) spin column according to the manufacturer’s instructions [47], followed by cDNA synthesis using High Capacity RNA-to-cDNA™ Kit (Fisher Scientific, Gothenburg, Sweden). Relative expression levels of the genes of interest as well as three housekeeping genes (mActin, β-Tubulin, and glycerylaldehyde 3-phosphate dehydrogenase or Gapdh) were determined with qPCR using MyiQ thermal cycler (Bio-Rad Laboratories, Solna, Sweden). Each reaction contained 5 ng/µL template, 20 mM Tris/HCl pH 9.0, 50 mM KCl, 4 mM MgCl_2_, 0.2 mM dNTP, (1:20) DMSO and (1:50,000) SYBR Green and 0.02 µg/mL Taq DNA polymerase [48]. All primers were designed using Beacon Design 8 (Premier Biosoft, Palo Alto, USA). For primers with annealing temperature ≥ 60 °C, initial 50 °C for 2 min, activation 95 °C for 2 min, denaturation at 95 °C for 15 s for 40 cycles and annealing/extend at 60 °C for 1 min was used for 40 cycles. For primers with annealing temperature ≤ 60 °C, initial 50 °C for 2 min, activation 95 °C for 2 min, denaturation at 95 °C for 15 sec for 40 cycles, annealing/extend at 55–60 °C for 15 s for 40 cycles and extend at 72 °C for 1 min of 40 cycles [18]. The relative amount of each transcript was determined using delta Ct values [49]. All reactions were performed in triplicates on each plate, and each plate was repeated twice. Negative controls were included on each plate.

To compare the transcript levels between different samples, three different methods were used as described below:

(1) 2-DDCt method was used [39], where the differences in the cycle threshold (Ct) values between the house keeping gene and a target gene, with or without treatment, was calculated. Then, the difference between these values was calculated as follows: Ct(treated) − Ct(non-treated) = (Ct(gene)-housekeeping) treated − (Ct(gene)-housekeeping) non-treated. To determine the ratio of expression levels in treated sample versus non-treated sample, the Qr formula was used as follows: Qr = 2 − Ct(treated) − Ct(non-treated).
(2) Efficiency-corrected Pfaffl Method was then performed. The fold difference is given by 1.85(A − B)/1.97(F − G) where A = average Cq of target gene in non-treated sample, B = average Cq of target gene in treated sample, F = average Cq of housekeeping reference gene in non-treated sample and G = average Cq of reference gene in treated sample. Primer efficiency value of the target gene is 1.85 while that of the the reference gene is 1.97 (both were computed according to LinReg).
(3) The third equation used the same equation as in two but instead of using the mean value, the minimum value was used to retrieve the lowest mean cycle threshold and then all quantities for this particular gene was expressed relative to this reaction. Finally, the graph was made using software GraphPad Prism 5.

### 4.6. Tissue Collection and Sectioning

All procedures involving mice (C57BL6/J mice; Taconic M&B, Ry, Denmark) were approved by the local ethical committee in Uppsala (permit numbers C39/16, C419/12, and C67/13), and were carried out according to the EU-directive 2010/63. All animals had ad libidum access to food and water unless stated otherwise. They were kept under a 12 h light–dark cycle and were sacrificed during the light period. Post intraperitoneal injection with pentobarbital (90 mg/kg IP; Apoteksbolaget, Stockholm, Sweden), trans-cardial perfusion was performed on adult male mice through the left ventricle with phosphate-buffered saline (PBS) followed by 4% formaldehyde (HistoLab, Gothenburg, Sweden). The brain was excised and stored in 4% formaldehyde overnight. The brain was then fixed in zinc-formalin (Richard-Allan Scientific, San Diego, CA, USA) for 18–24 h at 40 °C before dehydration and paraffin infusion (Tissue-Tek vacuum infiltration processor; Miles Scientific. Newark, CA, USA). The sections were cut (7 μm) using a Microm 355S STS cool cut microtome and attached on Superfrost Plus slides (Menzel-Gläser, Braunschweig, Germany). Then each slide was dried overnight at 37 °C and stored at 4 °C until use.

### 4.7. Fluorescent Immunohistochemistry on Paraffin Embedded Sections

Fluorescent immunohistochemistry was performed according to the procedures described in [9], with some exceptions. Sections were incubated with the commercial polyclonal antibody rabbit-anti-SLC38A6 (Sigma-Aldrich HPA018508) together with one of the antibody markers (NeuN, GFAP, and Pag) diluted in supermix (Tris-buffered saline, 0.25% gelatin, 0.5% Triton X-100) overnight at 4 °C (for antibody information see Table S2). After secondary antibody (tagged with Alexa Fluor 488 or 594) treatments (See Table S2 for concentrations) for 1 h and incubation with DAPI (Sigma-Aldrich, Stockholm, Sweden), the sections were mounted. 

PC12 cells were fixed using 4% para-formaldehyde after plating on coverslips. Immunostaining was performed using the antibodies and dilutions specified in Table S2 for 1 h. The samples were washed and incubated with the secondary antibodies and dilutions specified in the Table S2. 

Then sections were visualized using a Zeiss AxioPlan 2 fluorescence microscope (Zeiss, Jena, Germany), connected to an AxioCamHRm camera and the micrographs were finally analyzed with ImageJ [50] software.

Immuno-stained images in Figure 2A–C, Figure 3K–L and Figure 6A–C,G–I were acquired using a Zeiss LSM-700 confocal microscope using a × 63/1.40 objective (Zeiss, Jena, Germany) with sequential scanning of the red (excitation 561 nm, emission 578–696 nm) and green channel (excitation 488 nm, emission 493–574 nm). Pinhole size was 0.61 μm, corresponding to 1 Airy unit. Images were acquired in 16-bit at gain settings 750 for both channels. All the experiments with the microscopy techniques specified above were performed at least twice.

### 4.8. Plasmid Constructs 

Cav1-mCherry and Clathrin-mCherry was kindly provided by C Merrifield [51]. NPY-mCherry construct has been described elsewhere [52]. SNAT6 was generated by PCR amplification of a mouse brain cDNA library using primers forward 5-Nhe1-ATATATGCTAGCATGCAGGCGTCCCGGCAC 3′ and reverse -5′-AgeI-ATATATACCGGTTTTGTTGACCCAGTCAAA 3′. The PCR product was cloned in to NPY-EGFP construct using Age1 and Nhe1 restriction sites. PtdIns(4,5)P2 was detected using PH domains of PLCδ1-mRFP [53].

### 4.9. Proximity Ligation Assay (PLA)

The Duolink II fluorescence kit (orange detection reagents, Olink Biosciences, Uppsala, Sweden) was used to run in situ proximity ligation assay technology (PLA) on fixed cells and/or paraffin embedded sections according to manufacturer’s instructions [54,55,56,57]. The samples were blocked with blocking solution included in the kit for 30 min at 37 °C in a pre-heated humidity chamber. Specific primary antibodies (see supplement Table S2 for concentrations) diluted in antibody diluent included in the kit were added to the samples and incubated overnight at 4 °C in humid chamber. After that, PLA probes (PLUS and MINUS) were added for 1 h at 37 °C in the pre-heated humidity chamber. In our experiments, protein interactions were detected with combinations of anti-rabbit PLUS and anti-mouse MINUS or anti-mouse PLUS and anti-goat MINUS PLA probes. Then the detection protocol including ligation and amplification was followed. The ligation step, using ligase (provided in the kit) was performed for 30 min at 37 °C in pre-heated humidity chamber. The amplification step using polymerase (provided in the kit) was performed for 100 min at 37 °C in pre-heated humidity chamber. Then the slides were washed according to the manufacturer’s instructions with buffer provided in the kit, dried in the dark for 20 min and mounted using a minimal volume of Duolink In Situ Mounting Medium containing DAPI. The samples were then visualized using Zeiss Axioplan2 fluorescent microscope connected to AxioCamHRm camera. A negative control was included without primary antibodies. These experiments were performed twice independently. The images were further analyzed and quantified using Duolink ImageTool (Olink Biosciences, Uppsala, Sweden) software. 

### 4.10. TIRF Microscopy

All experiments, unless otherwise stated, were performed at 37 °C in an experimental buffer containing 125 mM NaCl, 4.9 mM KCl, 1.2 mM MgCl_2_, 1.3 mM CaCl_2_, 25 mM Hepes, 3 mM D-Glucose, and 0.1% BSA (pH 7.40). Amino acids including alanine, proline, arginine, serine, lysine, methionine, glutamate, and glutamine were added to the buffer in some of the experiments as stated in the Figures. NaCl was equimolarly replaced with KCl in experiments from Figure 4I–L (see Figure for details). Cells were imaged using a total internal reflection (TIRF) microscope based on an AxioObserver Z1 with a 100 ×/1.45 objective (Carl Zeiss, Jena, Germany). Excitation was from two DPSS lasers at 491 and 561 nm. The emission light was chromatically separated onto separate areas of an EMCCD camera (Photometrics Evolve) using an image splitter (Photometrics DV2, Photometrics, Tucson, AZ, USA). Scaling was maintained at 160 nm per pixel using ZEN blue (Carl Zeiss, Jena, Germany). Immuno-stained cells were imaged at 100 ms exposure with 561 (2 mW) and 491 (2 mW). SNAT6-GFP, 561 (0.5 mW) for Cav-mCherry cells were imaged at 100 ms per frame for static images and streams or movies with 491 (0.5 mW). Alignment of the two color channels was corrected as previously described [58].

### 4.11. Image Analysis

Six images per antibody-pair combination were taken from different areas of the brain using a Zeiss Axioplan 2 microscope, where 11 Z-stacks were acquired for each image. Filters suitable for the used fluorophores as well as a filter to detect auto fluorescence were utilized in order to deduct the background from auto fluorescence. The Z-stacked images were transformed using the maximum intensity projection function in the ImageJ software, v1.48 [59] to merge the signals into a one plane image. The CellProfiler software, v2.2.0 [60] was then used to analyze the signals. The auto-fluorescence was used to subtract background from the images, after which the images were cleared using a white tophat filter to remove anything over 10 pixels, leaving only the amplified signal. DAPI staining was used to define and identify cells by expanding the nuclei by 65 pixels. This was to enable automated counting of PLA signals within specific cells and all objects outside these specified regions were removed. The remaining signals with pixel intensity above 0.08 were automatically counted. The total signal was divided with number of cells to attain number of PLA signals per cell, before plotting using the GraphPad Prism 5 software. 

For TIRF experiments well separated SNAT6 clusters were identified that were far from the edge of the cell (at least seven per cell), and their locations transferred to the red channel. An algorithm implemented as MetaMorph (Molecular Devices, Sunnyvale, CA, USA) journal then read the average pixel fluorescence in (1) a central circle (c) of 3 pxl (0.5 μm) diameter, (2) a surrounding annulus (a) with an outer diameter of 5 pxl (0.8 μm) and (3) a background area not including any cell (bg). Since the site of interest is far smaller than the resolution of the microscope, the circle will contain all of the fluorescence originating from it. It also contains fluorescence from molecules not bound to the site of interest, which is estimated using “a”. To obtain the specific on-SNAT6 fluorescence ΔF, the annulus value (a) was therefore subtracted from that of the circle (c) (ΔF = c − a). To obtain off-SNAT6 fluorescence, the annulus value was background-corrected (S = a − bg). S represents the local unbound fluorescence, and averaged for each cell, S is linearly related to its expression level. The relationship of ΔF versus S follows a one-site binding equation that reaches saturation at higher expression levels. For relatively small S, the ratio ΔF/S is a convenient measure of binding to the SNAT6 site, which is independent of the expression level. Positive ΔF/S values indicate binding, negative values indicate exclusion (Gandasi and Barg, 2014). Independent percentage of co-localization was assessed by a journal in MetaMorph where it presented an observer (unaware of the image context) with square cutouts of the red channel (11 μm^2^) that were centered on the position of each previously identified SNAT6. The user then made a yes/no choice based on whether the center of the nearest perceived NPY/Clathrin/Caveolin was within one pixel of the center of the square, guided by an overlaid circle. The percentage of co-localized SNAT6 puncta was assessed using this method (Gandasi and Barg, 2014). Endocytosis events were identified based on the characteristic rapid loss of SNAT6 fluorescence within one or two frames. The fluorescence of cell was calculated by marking the cell and using the function measure intensities in Metamorph. Out of cell region was selected as background to calculate out from the cell fluorescence [52]. Cluster density was calculated using a script with the built-in “find maxima” function in ImageJ (http://rsbweb.nih.gov/ij) for spot detection (Gandasi and Barg, 2014). Tracking experiments were performed using a Metamorph tracking plugin with pixel size cut off = 5. The tracks obtained with X and Y co-ordinates were transferred to excel sheet and distance calculated per frame for each of the tracks. TIRF microscopy experiments were performed two to three times with at least eight cells per time per condition.

### 4.12. Uptake Assays Using Tritium Labeled Amino Acids 

PC1_2_ wildtype cells and a home-made (See method above) stable PC1_2_ cell line overexpressing SNAT6 were used for uptake assays with radio labeled amino acids. The cells were seeded in duplicates and were grown as described above in 48-well plates. The media was removed prior to the assays and 30 μL of 3H-labeled amino acids (glutamine, glutamate, alanine, proline, arginine, serine, lysine, and methionine) were added to the respective wells and incubated for 50 min and 70 min in two separate experiments. In total 0.2 μM of the labeled amino acid (hot) (PerkinElmer NEN^®^) and 100 μM of the non-labeled amino acid (cold) were diluted in KRH buffer containing 118 mM NaCl, 4.8 mM KCL, 1.2 mM MgSO_4_, 2.5 mM CaCl_2_, 10 mM HEPES depending on the specific activity of stock (Ci/mmol) and its concentration (μM). The final concentrations of the 3H-labeled L-amino acids were adjusted to 0.2 μM for each assay. Following washing on ice with ice cold KRH buffer the cells were lysed as above and were transferred to tubes containing 2.5 mL scintillation liquid. Finally, they were read with Quanta Smart software in the Tri-Carb B2910TR liquid scintillation analyzer measured as counts per min (cpm). These experiments were repeated twice to check for repeatability. The statistical analysis and graphs were made using software GraphPad Prism 5.

### 4.13. Statistics

All the error bars are presented with standard error of the mean (SEM) unless specified otherwise. All the results obtained were plotted using excel and Origin 8. Statistical analysis and calculations (Figure 1 and Figure 3) have been done using software GraphPad Prism 5. One-way ANOVA was used for calculations concerning the uptake assays. Statistical analysis was performed using Origin 8. Unpaired *t*-tests with 95% confidence interval was performed between different treatment groups (* *p* < 0.05, ** *p* < 0.01, *** *p* < 0.001). See the figure legends for details.

## Data Availability

Details of all experiments including data and material, performed for this article, will be made accessible. They are either included in the manuscript or are available on request.

## Supplementary Materials

The following are available online at https://www.mdpi.com/1422-0067/22/3/1167/s1, Table S1—listing the candidate genes described in Figure 1E. Table S2. Details of antibodies used for fluorescent immunohistochemistry and Proximity Ligation Assay.

## References

[1] Almén M.S., Nordström K.J.V., Fredriksson R., Schiöth H.B. (2009). Mapping the human membrane proteome: A majority of the human membrane proteins can be classified according to function and evolutionary origin. BMC Biol..

[2] Perland E., Fredriksson R. (2017). Classification Systems of Secondary Active Transporters. Trends Pharmacol. Sci..

[3] Rask-Andersen M., Masuram S., Fredriksson R., Schiöth H.B. (2013). Solute carriers as drug targets: Current use, clinical trials and prospective. Mol. Asp. Med..

[4] César-Razquin A., Snijder B., Frappier-Brinton T., Isserlin R., Gyimesi G., Bai X., Reithmeier R.A.F., Hepworth D., Hediger M.A., Edwards A.M. (2015). A Call for Systematic Research on Solute Carriers. Cell.

[5] Willard S.S., Koochekpour S. (2013). Glutamate Signaling in Benign and Malignant Disorders: Current Status, Future Perspectives, and Therapeutic Implications. Int. J. Biol. Sci..

[6] Erickson J.D. (2017). Functional identification of activity-regulated, high-affinity glutamine transport in hippocampal neurons inhibited by riluzole. J. Neurochem..

[7] Lichter-Konecki U. (2008). Profiling of astrocyte properties in the hyperammonaemic brain: Shedding new light on the pathophysiology of the brain damage in hyperammonaemia. J. Inherit. Metab. Dis..

[8] Albrecht J., Sidoryk-Węgrzynowicz M., Zielińska M., Aschner M. (2010). Roles of glutamine in neurotransmission. Neuron Glia Biol..

[9] Bagchi S., Baomar H.A., Al-Walai S., Al-Sadi S., Fredriksson R. (2014). Histological Analysis of SLC38A6 (SNAT6) Expression in Mouse Brain Shows Selective Expression in Excitatory Neurons with High Expression in the Synapses. PLoS ONE.

[10] Todd A.C., Marx M.-C., Hulme S.R., Bröer S., Billups B. (2017). SNAT3-mediated glutamine transport in perisynaptic astrocytesin situis regulated by intracellular sodium. GLIA.

[11] González M.I., Krizman-Genda E., Robinson M.B. (2007). Caveolin-1 Regulates the Delivery and Endocytosis of the Glutamate Transporter, Excitatory Amino Acid Carrier 1. J. Biol. Chem..

[12] Bröer S. (2013). The SLC38 family of sodium–amino acid co-transporters. Pflüg. Archiv Eur. J. Physiol..

[13] Rae C.D., Hare N., Bubb W.A., McEwan S.R., Bröer A., McQuillan J.A., Balcar V.J., Conigrave A.D., Bröer S. (2003). Inhibition of glutamine transport depletes glutamate and GABA neurotransmitter pools: Further evidence for metabolic compartmentation. J. Neurochem..

[14] Conti F., Melone M. (2006). The glutamine commute: Lost in the tube?. Neurochem. Int..

[15] Bansal M., Belcastro V., Ambesi-Impiombato A., Di Bernardo D. (2007). How to infer gene networks from expression profiles. Mol. Syst. Biol..

[16] Margolin A.A., Nemenman I., Basso K., Wiggins C., Stolovitzky G., Dalla-Favera R., Califano A. (2006). ARACNE: An Algorithm for the Reconstruction of Gene Regulatory Networks in a Mammalian Cellular Context. BMC Bioinform..

[17] Van Someren E., Wessels L., Backer E., Reinders M. (2002). Genetic network modeling. Pharmacogenomics.

[18] Hellsten S.V., Lekholm E., Ahmad T., Fredriksson R. (2017). The gene expression of numerous SLC transporters is altered in the immortalized hypothalamic cell line N25/2 following amino acid starvation. FEBS Open Bio.

[19] Butchbach M.E., Tian G., Guo H., Lin C.-L.G., Tzameli I., Fang H., Ollero M., Shi H., Hamm J.K., Kievit P. (2004). Association of Excitatory Amino Acid Transporters, Especially EAAT2, with Cholesterol-rich Lipid Raft Microdomains. J. Biol. Chem..

[20] Murphy-Royal C., Dupuis J.P., Varela J.A., Panatier A., Pinson B., Baufreton J., Groc L., Oliet S.H.R. (2015). Surface diffusion of astrocytic glutamate transporters shapes synaptic transmission. Nat. Neurosci..

[21] Head B.P., Insel P.A. (2007). Do caveolins regulate cells by actions outside of caveolae?. Trends Cell Biol..

[22] Omasits U., Ahrens C.H., Müller S., Wollscheid B. (2014). Protter: Interactive protein feature visualization and integration with experimental proteomic data. Bioinformatics.

[23] Biasini M., Bienert S., Waterhouse A., Arnold K., Studer G., Schmidt T., Kiefer F., Cassarino T.G., Bertoni M., Bordoli L. (2014). SWISS-MODEL: Modelling protein tertiary and quaternary structure using evolutionary information. Nucleic Acids Res..

[24] Arnold K., Bordoli L., Kopp J., Schwede T. (2005). The SWISS-MODEL workspace: A web-based environment for protein structure homology modelling. Bioinformatics.

[25] Benkert P., Biasini M., Schwede T. (2010). Toward the estimation of the absolute quality of individual protein structure models. Bioinformatics.

[26] Hellsten S.V., Hägglund M.G., Eriksson M.M., Fredriksson R. (2017). The neuronal and astrocytic protein SLC38A10 transports glutamine, glutamate, and aspartate, suggesting a role in neurotransmission. FEBS Open Bio.

[27] Gandasi N., Barg S. (2014). Contact-induced clustering of syntaxin and munc18 docks secretory granules at the exocytosis site. Nat. Commun..

[28] Schiöth H.B., Roshanbin S., Hägglund M.G., Fredriksson R. (2013). Evolutionary origin of amino acid transporter families SLC32, SLC36 and SLC38 and physiological, pathological and therapeutic aspects. Mol. Asp. Med..

[29] Omar-Hmeadi M., Gandasi N., Barg S. (2018). PtdIns(4,5)P2 is not required for secretory granule docking. Traffic.

[30] Xie B., Nguyen P.M., Guček A., Thonig A., Barg S., Idevall-Hagren O. (2016). Plasma Membrane Phosphatidylinositol 4,5-Bisphosphate Regulates Ca^2+^ -Influx and Insulin Secretion from Pancreatic β Cells. Cell Chem. Biol..

[31] Crupi R., Impellizzeri D., Cuzzocrea S. (2019). Role of Metabotropic Glutamate Receptors in Neurological Disorders. Front. Mol. Neurosci..

[32] Perl K., Ushakov K., Pozniak Y., Yizhar-Barnea O., Bhonker Y., Shivatzki S., Geiger T., Avraham K.B., Shamir R. (2017). Reduced changes in protein compared to mRNA levels across non-proliferating tissues. BMC Genom..

[33] Samuvel D.J., Jayanthi L.D., Bhat N.R., Ramamoorthy S. (2005). A Role for p38 Mitogen-Activated Protein Kinase in the Regulation of the Serotonin Transporter: Evidence for Distinct Cellular Mechanisms Involved in Transporter Surface Expression. J. Neurosci..

[34] Fecchi K., Volonte D., Hezel M.P., Schmeck K., Galbiati F. (2006). Spatial and temporal regulation of GLUT4 translocation by flotillin-1 and caveolin-3 in skeletal muscle cells. FASEB J..

[35] Underhill S.M., Wheeler D.S., Li M., Watts S.D., Ingram S.L., Amara S.G. (2014). Amphetamine Modulates Excitatory Neurotransmission through Endocytosis of the Glutamate Transporter EAAT3 in Dopamine Neurons. Neuron.

[36] Menchini R.J., Chaudhry F.A. (2019). Multifaceted regulation of the system A transporter Slc38a2 suggests nanoscale regulation of amino acid metabolism and cellular signaling. Neuropharmacology.

[37] Alshammari M.A., Alshammari T.K., Laezza F. (2016). Improved Methods for Fluorescence Microscopy Detection of Macromolecules at the Axon Initial Segment. Front. Cell. Neurosci..

[38] Lamberts R., Goldsmith P.C. (1986). Fixation, fine structure, and immunostaining for neuropeptides: Perfusion versus immersion of the neuroendocrine hypothalamus. J. Histochem. Cytochem..

[39] Tawfik D., Zaccagnino A., Bernt A., Szczepanowski M., Klapper W., Schwab A., Kalthoff H., Trauzold A. (2020). The A818–6 system as an in-vitro model for studying the role of the transportome in pancreatic cancer. BMC Cancer.

[40] Ye J., Xu B., Fan B., Zhang J., Yuan F., Chen Y., Sun Z., Yan X., Song Y., Song S. (2020). Discovery of Selenocysteine as a Potential Nanomedicine Promotes Cartilage Regeneration with Enhanced Immune Response by Text Mining and Biomedical Databases. Front. Pharmacol..

[41] Grainger A.T., Jones M.B., Chen M.-H., Shi W. (2017). Polygenic Control of Carotid Atherosclerosis in a BALB/cJ × SM/J Intercross and a Combined Cross Involving Multiple Mouse Strains. G3 Genes Genomes Genet..

[42] Guex N., Peitsch M.C. (1997). SWISS-MODEL and the Swiss-Pdb Viewer: An environment for comparative protein modeling. Electrophoresis.

[43] Irizarry R.A., Hobbs B., Collin F., Beazer-Barclay Y.D., Antonellis K.J., Scherf U., Speed T.P. (2003). Exploration, normalization, and summaries of high density oligonucleotide array probe level data. Biostatistics.

[44] Li C. (2001). Model-based analysis of oligonucleotide arrays: Expression index computation and outlier detection. Proc. Natl. Acad. Sci. USA.

[45] Johnson D.E., Ai H.-W., Wong P., Young J.D., Campbell R.E., Casey J.R. (2009). Red Fluorescent Protein pH Biosensor to Detect Concentrative Nucleoside Transport. J. Biol. Chem..

[46] Shannon P., Markiel A., Ozier O., Baliga N.S., Wang J.T., Ramage D., Amin N., Schwikowski B., Ideker T. (2003). Cytoscape: A Software Environment for Integrated Models of Biomolecular Interaction Networks. Genome Res..

[47] Rihani A., Van Goethem A., Ongenaert M., De Brouwer S., Volders P.-J., Agarwal S., De Preter K., Mestdagh P., Shohet J., Speleman F. (2015). Genome wide expression profiling of p53 regulated miRNAs in neuroblastoma. Sci. Rep..

[48] Williams M.J., Goergen P., Rajendran J., Zheleznyakova G.Y., Hägglund M.G., Perland E., Bagchi S., Kalogeropoulou A., Khan Z., Fredriksson R. (2014). Obesity-Linked Homologues TfAP-2 and Twz Establish Meal Frequency in Drosophila melanogaster. PLoS Genet..

[49] Pfaffl M.W. (2001). A new mathematical model for relative quantification in real-time RT-PCR. Nucleic Acids Res..

[50] Schneider C.A., Rasband W.S., Eliceiri K.W. (2012). NIH Image to ImageJ: 25 years of image analysis. Nat. Methods.

[51] Taylor M.J., Perrais D., Merrifield C.J. (2011). A High Precision Survey of the Molecular Dynamics of Mammalian Clathrin-Mediated Endocytosis. PLoS Biol..

[52] Gandasi N., Vestö K., Helou M., Yin P., Saras J., Barg S. (2015). Survey of Red Fluorescence Proteins as Markers for Secretory Granule Exocytosis. PLoS ONE.

[53] Stauffer T.P., Ahn S., Meyer T. (1998). Receptor-induced transient reduction in plasma membrane PtdIns(4,5)P2 concentration monitored in living cells. Curr. Biol..

[54] Jarvius M., Paulsson J., Weibrecht I., Leuchowius K.-J., Andersson A.-C., Wählby C., Gullberg M., Botling J., Sjöblom T., Markova B. (2007). In SituDetection of Phosphorylated Platelet-derived Growth Factor Receptor β Using a Generalized Proximity Ligation Method. Mol. Cell. Proteom..

[55] Söderberg O., Gullberg M., Jarvius M., Ridderstråle K., Leuchowius K.-J., Jarvius J., Wester K., Hydbring P., Bahram F., Larsson L.-G. (2006). Direct observation of individual endogenous protein complexes in situ by proximity ligation. Nat. Methods.

[56] Gullberg M., Gústafsdóttir S.M., Schallmeiner E., Jarvius J., Bjarnegård M., Betsholtz C., Landegren U., Fredriksson S. (2004). Cytokine detection by antibody-based proximity ligation. Proc. Natl. Acad. Sci. USA..

[57] Fredriksson S., Gullberg M., Jarvius J., Olsson C., Pietras K., Gústafsdóttir S.M., Östman A., Landegren U. (2002). Protein detection using proximity-dependent DNA ligation assays. Nat. Biotechnol..

[58] Taraska J.W., Perrais D., Ohara-Imaizumi M., Nagamatsu S., Almers W. (2003). Secretory granules are recaptured largely intact after stimulated exocytosis in cultured endocrine cells. Proc. Natl. Acad. Sci. USA.

[59] Kamentsky L., Jones T.R., Fraser A., Bray M.-A., Logan D.J., Madden K.L., Ljosa V., Rueden C., Eliceiri K.W., Carpen-ter A.E. (2011). Improved structure, function and compatibility for CellProfiler: Modular high-throughput image analysis software. Bioinformatics.

[60] Carpenter A.E., Jones T.R., Lamprecht M.R., Clarke C., Kang I.H., Friman O., Guertin D.A., Chang J.H., Lindquist R.A., Moffat J. (2006). CellProfiler: Image analysis software for identifying and quantifying cell phenotypes. Genome Biol..

