# Supplementary material for: Glutamine Uptake via SNAT6 and Caveolin Regulates Glutamine–Glutamate Cycle
Source: Int J Mol Sci. 2021 Jan 25;22(3):1167. doi: 10.3390/ijms22031167 (PMC7865731; doi:10.3390/ijms22031167)
Supplement: Supplementary file 1 [file ijms-22-01167-s001.pdf]

genes list
Alg1
Aipl1
Ankrd45
Anks1b
Apba3
Arpc2
Ascl3
Asgr1
Atad2b
Atp2b4
Ccana1g
CD28
Cdk4
Clec10a
Cog5
Creb313 (Cre
Ctps2
Daam1
Dlp2a
Dnach2
Dock10
Dnmt3a
Drdc
Eml6
EpB4.115(EP
Erbb4
Fabp3
Farp2
Flii
Fn1
Gabra6
Gja1
Grm2
Gp49a
Grin3b
Ints7
Itm2c
Krt20
Lactb2
Lmbrd1
Lrfn5
Mar-10
Mdm2
Mfsd12
Mks1
Mum1
Nab1
Ndn
Nt5c3l
Olfr218
P2rx1
Pag
Phlpp1
Pik3c2b
Pkhd1
Prex2
Ptpn14
Ranbp2
Rnaseh1

Sez6
Sirt7
Slamf8
Slc38a6
Slc5a10
Slit3
Snat10
Sox13
Spog
Srebf1
Stk36
Syne1
Taco1
Tbc1d15
Tex14
Tha1
Tie2
Tmem131
Tnfrs11a
Uhrf1bp1l
Zfp354a
Zmiz2

Supplementary table 1

**Primary antibodies**	**Species**	**Dilution**	**Supplier**
CTPs2	Rabbit	1:200	Innovagen
NeuN	Mouse	1:400	Millipore
GFAP	Mouse	1:400	Millipore
Pag	Mouse	1:100	AbCam
Synaptophysin	Mouse	1:100	BD Biosciences
Grm2	Mouse	1:100	AbCam
SNAT6	Rabbit	1:100	Sigma
Pag	Mouse	1:100	Sigma
CTPs2	Rabbit	1:200	Sigma
Sytnaptophysin	Rat	1:100	Milipore
Caveolin	Mouse	1:200	Sigma
**Secondary antibodies**	**Species**	**Dilution**	**Supplier**
Anti-mouse-488	Goat	1:400	Invitrogen
Anti-rabbit-594	Donkey	1:400	Invitrogen
Anti-rabbit-488	Donkey	1:200	Jackson Immuno Reserach
Anti-mouse-594	Donkey	1:200	Jackson Immuno Reserach
Anti-mouse-488	Donkey	1:200	Invitrogen

Supplementary table 2
